# Local and Global Limits on Visual Processing in Schizophrenia

**DOI:** 10.1371/journal.pone.0117951

**Published:** 2015-02-17

**Authors:** Marc S. Tibber, Elaine J. Anderson, Tracy Bobin, Patricia Carlin, Sukhwinder S. Shergill, Steven C. Dakin

**Affiliations:** 1 Institute of Ophthalmology, University College London, London, United Kingdom; 2 UCL Institute of Cognitive Neuroscience, London, United Kingdom; 3 Institute of Psychiatry, Psychology and Neuroscience, King’s College London, London, United Kingdom; 4 Department of Forensic and Neurodevelopmental Science, Institute of Psychiatry, Psychology and Neuroscience, King’s College London, London, United Kingdom; 5 Department of Optometry & Vision Science, University of Auckland, Private Bag, Auckland, New Zealand; Ecole Polytechnique Federale de Lausanne, SWITZERLAND

## Abstract

Schizophrenia has been linked to impaired performance on a range of visual processing tasks (e.g. detection of coherent motion and contour detection). It has been proposed that this is due to a general inability to integrate visual information at a global level. To test this theory, we assessed the performance of people with schizophrenia on a battery of tasks designed to probe voluntary averaging in different visual domains. Twenty-three outpatients with schizophrenia (mean age: 40±8 years; 3 female) and 20 age-matched control participants (mean age 39±9 years; 3 female) performed a motion coherence task and three equivalent noise (averaging) tasks, the latter allowing independent quantification of *local* and *global* limits on visual processing of motion, orientation and size. All performance measures were indistinguishable between the two groups (*ps*>0.05, one-way ANCOVAs), with one exception: participants with schizophrenia pooled fewer estimates of local orientation than controls when estimating average orientation (*p* = 0.01, one-way ANCOVA). These data do not support the notion of a generalised visual integration deficit in schizophrenia. Instead, they suggest that distinct visual dimensions are differentially affected in schizophrenia, with a specific impairment in the integration of visual orientation information.

## Introduction

Schizophrenia (SZ) is a mental disorder characterised by cognitive, affective and perceptual symptoms including anomalous visual processing (see [1] for a review). Thus, people with SZ perform differently from unaffected controls on a range of visual tasks, from simple texture discrimination in the presence of a suppressive surround [2–4], to more complex ‘high-level’ processes such as face perception [5,6] and the detection of biological motion [7]. One hypothesis that attempts to link these seemingly disparate findings is that SZ is characterised by a relative inability to integrate (or bind) visual information at a global level [8], such that perception is fragmented [9]. Consistent with this theory, observers with SZ typically perform poorly on tasks in which local features must be integrated to reveal global form [10,11] or global motion [12,13]. For example, participants with SZ are less accurate than controls at indicating the location of a contour composed of a chain of discrete oriented elements (Gabors) embedded in an array of randomly oriented distracters (a *contour detection* paradigm) [14–18]. Similarly, participants with SZ require a higher percentage of dots to be drifting in the same direction for the predominant (global) direction of motion to be reported in a random-dot stimulus (a *motion coherence* paradigm) [13,19–21].

Participants with SZ also exhibit a number of visuoperceptual abnormalities that are not so readily reconciled with impaired perceptual integration, e.g. reduced contrast sensitivity [22,23] and impaired velocity discrimination [24–29]. Further, performance on ‘global integration’ tasks traditionally used in studies of SZ may not be limited solely by the participant’s ability to integrate information. For example, performance on motion coherence tasks may also be limited by noisy (i.e. imprecise) encoding of local directions, or an inability to exclude noise [30,31]; impaired processing of faces and biological motion in SZ has recently been linked to deficits in the encoding of local stimulus features [32,33]; and elevated contour detection thresholds in SZ may also be limited by imprecise encoding of individual orientations [34] or abnormal contextual effects operating over a relatively short distance [34,35].

Thus, there is still considerable uncertainty as to whether poor performance on ‘global integration’ tasks in SZ truly reflects an integration deficit. Previous studies do not speak to this hypothesis directly, since they typically employ tasks that are limited -and therefore confounded- by local *and* global processing. To disentangle these factors we used a psychophysical paradigm known as equivalent noise (EN), which allows performance on a global averaging task to be parcellated into *independent* estimates of local processing (internal noise) and global processing (sampling) [36]. Twenty-three participants with SZ and 20 age-matched controls were tested on a standard motion coherence paradigm and three versions of the EN paradigm, the latter separately quantifying local and global limits to judgements of average motion, average orientation and average size [37]. Specifically, we tested the hypothesis that impaired perceptual integration represents a generalised characteristic of visual processing in SZ. We predicted that relative to control participants, those with SZ would exhibit elevated motion coherence thresholds and lower levels of sampling for all three visual dimensions.

## Materials and Methods

Ethics approval was obtained for this study from the UK National Research Ethics Committee. In accordance with the declaration of Helsinki informed written consent was obtained from each participant.

### Participants

Data were gathered from 23 participants with SZ (three female) and 20 healthy control participants (three female) (CON) (Table 1). The two groups did not differ significantly with respect to age [mean score: 40±8 (SZ) and 38±9 years (CON); t_(41)_ = -0.81, *p* = 0.43; Cohen’s d = 0.25]. Participants with SZ were recruited from outpatients at the Institute of Psychiatry (IoP); all had been diagnosed with SZ according to DSM-IV-R criteria by a Masters level research nurse with extensive knowledge and training in the field. Since this diagnosis excludes anybody with schizoaffective disorder or mood disorder with psychotic features, participants with affective psychosis were not included in the study. Of the 23 patients tested, 13 were diagnosed with paranoid SZ; none of the other patients fell firmly into any other specific sub-category. Participants’ symptom severity was assessed using the Positive and Negative Symptoms Scale (PANSS) [38] within one week of psychophysical testing. None of the control participants had a history of mental illness. All participants had a minimum visual acuity of 20/20 binocularly (with or without optometric correction).

**Table 1:** Clinical data for the participants with schizophrenia.

												
Diag	Sex	Age	Med	Type	Dose	IQ	tPANSS	tPSS	tNSS	tGSS	tDIS	DIS
SZ	M	39	Aripiprazole	2nd	133	95	44	9	12	23	9	1
PS	M	30	Clozapine	2nd	1000	106	58	12	20	26	9	1
PS	M	30	Olanzapine	2nd	400	102	47	7	17	23	10	1
SZ	F	38	Clozapine	2nd	800	100	100	20	28	52	15	4
SZ	M	40	Risperidone	2nd	100	106	76	14	30	32	14	3
PS	M	49	Haloperidol	1st	200	98	48	13	11	24	9	2
SZ	M	33	Olanzapine	2nd	400	105	69	14	20	35	9	3
PS	M	48	Olanzapine	2nd	400	100	67	18	21	28	10	1
PS	M	42	Clozapine	2nd	750	101	59	13	14	32	9	1
SZ	M	31	Quetiapine	2nd	1066	112	61	15	16	30	11	2
SZ	F	50	Pipotiazine	1st	150	112	61	21	11	29	12	3
PS	M	53	Clozapine	2nd	1000	89	40	12	9	19	8	2
SZ	M	36	Olanzapine	2nd	200	111	42	7	14	21	8	1
PS	M	43	Clozapine	2nd	1200	86	63	15	16	32	9	3
PS	M	28	Pipotiazine	1st	200	101	64	11	23	30	14	3
PS	M	46	Clozapine	2nd	600	117	47	8	12	27	8	1
SZ	M	53	-	-	150	95	73	16	25	32	11	1
PS	M	28	Clozapine	2nd	500	84	53	9	20	24	14	3
SZ	M	31	Clozapine	2nd	800	100	63	13	18	32	10	1
PS	M	45	Olanzapine	2nd	400	105	65	17	19	29	12	3
PS	F	43	Quetiapine	2nd	1400	117	55	12	17	26	11	2
PS	M	40	Clozapine	2nd	300	113	34	7	11	16	5	1
SZ	M	45	Olanzapine	2nd	200	94	47	12	12	23	8	1
Mean	-	40	-	-	537	102	58	13	17	28	10	2
Stdev	-	8	-	-	389	9	14	4	6	7	2	1

The following information is provided: diagnosis (Diag; SZ = schizophrenia; PS = Paranoid schizophrenia), medication (Med), medication type (Type: 1st = first generation antipsychotic; 2nd = second generation antispsychotic), medication dose (Dose: chlorpromazine equivalent in mg/day), intelligence quotient (IQ / NART score), total scores for the entire PANSS test (tPANSS), total scores for the positive symptoms of the PANSS test (tPSS), total scores for the negative symptoms of the PANSS test (tNSS), total scores for the general symptoms of the PANSS test (tGSS), scores on a cognitive factor which overlaps heavily with the concept of disorganization syndrome (tDIS) and scores for item P2 on the PANSS test, “conceptual disorganization” (DIS).

### General procedure

The experiment lasted approximately 90 minutes and consisted of: (i) a test of visual acuity (LogMar near visual acuity chart); (ii) a rapid assessment of IQ (National Adult Reading Test; NART) [39]; (iii) assessment of motion coherence thresholds; (iv) three EN experiments, probing orientation, motion and size processing. Psychophysical tasks were blocked and presented in a random order. Responses were given verbally and relayed to the computer by the experimenter.

### Motion Coherence procedure

**Fig 1:**
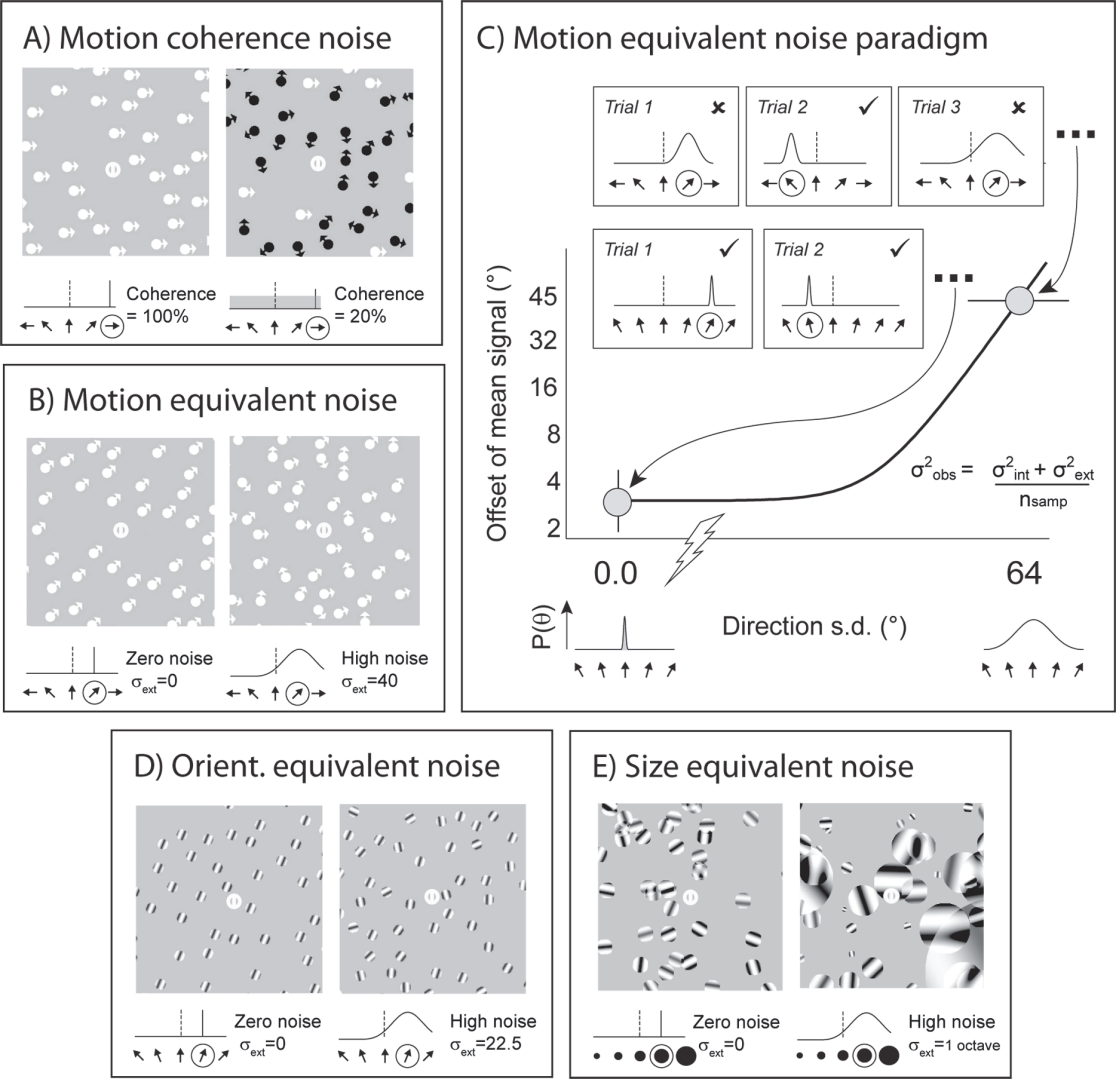
Psychophysical procedures. (A) Example high (100%) and low (20%) coherence motion stimuli. Signal dots are shown in white and noise dots in black. Directions of motion are indicated by the orientation of the arrow-heads. (Note: in the actual experiment all dots were white). Below each example stimulus is shown the corresponding idealised distribution of signal values (solid black line) and noise values (dark grey shaded region). In the coherence task, noise was increased by changing the proportion of signal to noise dots. (B) Zero and high noise motion stimuli, with corresponding distributions of motion directions presented below. (C) The equivalent noise fit (solid black line) is constrained by two data-points: the ‘zero noise’ threshold, which represents the minimum directional offset that can be reliably discriminated, and the ‘high noise’ threshold, which represents the maximum level of noise that can be tolerated while discriminating between large directional offsets (±45°). The fitting-function (inset in C) has two parameters: internal noise and global sampling. (D) and (E) show zero and high noise orientation and size stimuli, for orientation and size judgements, respectively. The schematics below show corresponding distributions of orientations / sizes. In (A, B, D & E), the reference direction / orientation / size is denoted by a vertical black dotted line; the average signal direction / orientation / size is circled.

Full details of the psychophysical methods used are given elsewhere [40,41]. In brief, participants reported the direction of motion of a variable number of coherently moving signal-dots embedded in noise (dots moving in random directions). Signal dots were restricted to motion in the horizontal plane (all-left or all-right on any given trial). Noise was added by assigning a subset of dots random directions of motion (Fig. 1A). An adaptive staircase procedure (QUEST; [42]) manipulated the level of coherence on each trial (the percentage of dots that constituted the signal), such that it converged on the 82% (correct) coherence threshold. The staircase terminated after 75 trials. Prior to the start of the testing phase all participants completed 15 practice trials in order to familiarise themselves with the nature of the task.

### Equivalent noise procedure

An efficient version of the EN paradigm was used to assess local and global processing limits [40]. As in previous applications of EN to this problem, observers performed a series of *voluntary averaging* tasks, judging whether stimulus elements were, on average, drifting clockwise or anti-clockwise of vertical-upward motion (motion task; Fig. 1B), tilted to the left or right of vertical (orientation task; Fig. 1D), or smaller or larger than a reference (size task; Fig. 1E). The reference direction, orientation and size were defined within the fixation marker, which was a small white circle bisected by a vertical line.

Two independent QUEST staircases were randomly interleaved (Fig. 1C): in the ‘zero noise’ condition, external noise was set to zero (i.e. all elements drifted in the same direction, were iso-oriented or were equal in size) and QUEST tracked the minimum directional-offset from vertical, orientation-offset from vertical, or size-offset from reference supporting reliable (82% correct) discrimination performance (motion, orientation and size tasks, respectively). For example, in the motion task, the directional-offset of all elements varied from trial to trial as a function of the participant’s responses: if the participant reported the direction of motion *correctly* (Fig. 1C, Trial 1, lower inlays), the size of the offset in the next trial *decreased*, such that the judgement became harder (Fig. 1C, Trial 2, lower inlays). Conversely, if the participant reported the direction of motion *incorrectly*, the size of the offset subsequently *increased* on the next trial, such that the judgement became easier. Under the control of QUEST, this process was repeated across the block of trials, such that the directional-offset presented eventually stabilised about the participant’s threshold offset (i.e. the minimum stimulus offset required for the participant to accurately report its direction of motion on a given proportion of trials). In the ‘high noise’ condition, the staircase tracked the maximum level of signal variability (external noise) that could be tolerated for observers to discriminate which of two possible, large signal-offsets (fixed at ±45°, ±22.5°and ±0.5 octaves for the motion, orientation and size tasks, respectively) were present (maximum tolerable noise). For example, in the motion task, the mean direction of motion was fixed at 45°CW or ACW of vertical; however, on each trial, the standard deviation of directions presented varied as a function of the participant’s responses. Thus, if the observer reported the mean direction of motion *incorrectly* on a given trial (Fig. 1C, trial 1, upper inlays), the standard deviation of directions present in the stimulus (i.e. noise added) was *decreased* on the next trial (Fig. 1C, trial 2, upper inlays), such that the judgement was made easier. Conversely, if the participant reported the predominant direction of motion *correctly* (Fig. 1C, trial 2, upper inlays), the noise added to the stimulus on the next trial was *increased*, such that the judgement was made harder (Fig. 1C, trial 3, upper inlays). Under the control of QUEST, this process was repeated across the block of trials, such that the standard deviation of the stimulus stabilised about the threshold level of noise that could be tolerated by the participant (i.e. the maximum standard deviation of directions in the stimulus that could be tolerated whilst the participant accurately reported the mean direction of motion for a given proportion of trials). Both staircases terminated after 75 trials (each). A two-parameter EN function was then fit to each participant’s data (Fig. 1C), providing estimates of internal noise (a measure of local processing) and sampling (a measure of global processing). (See [40] for full details). All test blocks (one per task type) were preceded by 15 practice trials in order to familiarise the participant with the nature of the task.

### Stimulus parameters

Stimuli were generated in Matlab (MathWorks, Cambridge, MA) with the Psychophysics Toolbox extensions [43,44] and were presented on the built-in LCD display of a MacBook Pro laptop computer (resolution of 1280x800 pixels at60Hz). Test images were comprised of 100 elements dropped within a circular region with a diameter of 15°. In the motion task, overlapping elements led to occlusion. In the size task, the contrasts of overlapping elements were summed. Element overlap was unavoidable in these versions of the task because of the basic physical constraints of presenting a high number of elements (varying in size or direction of motion) within a small region of the visual field. In contrast, for the orientation task, a minimum centre-to-centre spacing of elements (equal to the element- diameter) ensured that adjacent elements could *not* overlap. This was deemed appropriate since overlapping elements that occluded one another would have led to a loss of orientation information, whist overlapping elements with partial transparency would have generated orientation artifacts (i.e. plaids). Images were presented at screen-centre for 400 milliseconds against a grey background, and were viewed in a dark room at a distance of 51cm. The fixation marker had a diameter of 0.44°.

For the orientation task, individual elements comprised random phase sine-wave gratings (spatial frequency = 3.4 cycles per degree, presented at 50% contrast) windowed by a circular hard-edged mask with a diameter of 0.44°. Disks were similar for the size task, but varied in size and were randomly oriented. The spatial frequency of the carrier-grating was always scaled relative to the diameter of the disk so that the number of cycles presented was constant across changes in size. In addition, for the size task, the contrast of individual disks was randomly jittered (between 25–75%) to minimise any cues from contrast-differences. For the motion tasks, individual elements were comprised of white dots with a diameter of 0.44° presented at 50% contrast (Fig. 1B); these had a lifetime of 300ms, drifted at 3°/sec and their position was updated every 50ms.

### Data transformation and filtration

Data were analysed as described previously [40], facilitating directing comparison between data-sets. In brief, all psychophysical data were log-transformed as this reduced skew and kurtosis. Following log-transformation, the distribution of variables did not differ significantly from normal (*ps*>0.05; one-sample Kolmogorov-Smirnoff tests). Data were then filtered (separately for CON and SZ groups) so that extreme outliers with respect to parameter estimates and associated confidence intervals (>2.58 Z-scores from the group mean) were excluded from analyses. This led to the exclusion of 4.7% of the data. Although the filtration process had negligible effects on the findings, we also present statistics undertaken on non-filtered (i.e. raw) data for key comparisons, as well as with and without IQ built into the model as a covariate. In addition, raw and filtered psychophysical data are presented in S1 Dataset; these include basic offset thresholds and maximum tolerable noise levels, as well as levels of internal noise and sampling.

## Results

A series of independent t-tests indicated that none of the psychophysical measures recorded differed significantly (*p*>0.05) between SZ sub-groups (paranoid vs. non-paranoid schizophrenia); nor did these two SZ sub-groups differ with respect to age, IQ, medication dose (chlorpromazine equivalents) or PANSS scores (general, positive or negative). Consequently, data from all observers with SZ were pooled for subsequent analyses. Since IQ levels were found to be significantly lower in the participants with SZ than those without (t_(38)_ = 2.1, *p* = 0.04, Cohen’s d = 0.69), analyses were run both with and without IQ scores included as a covariate. IQ scores were available for 40 out of the 43 participants.

### Motion coherence thresholds

**Fig 2:**
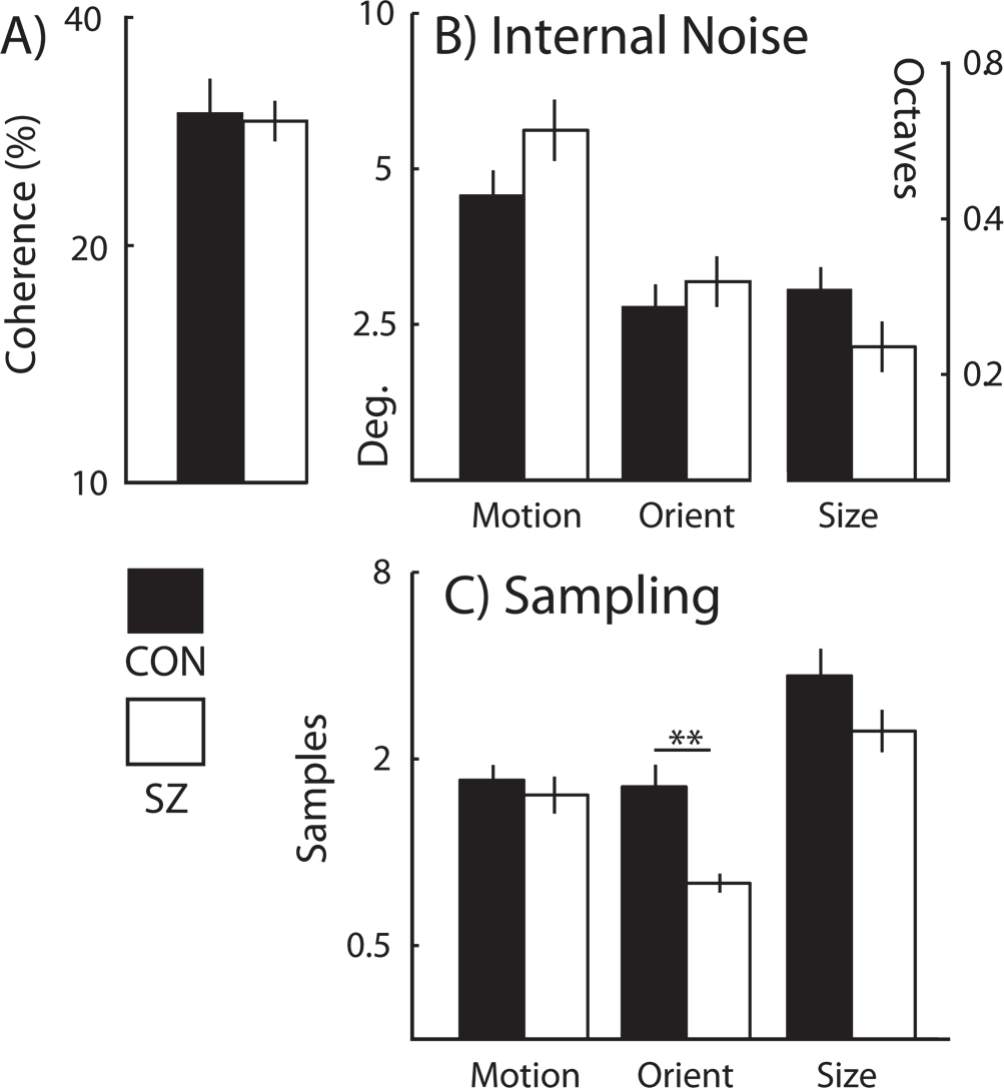
Coherence and equivalent noise plots. Group mean (A) coherence thresholds, (B) levels of internal noise and (C) sampling are shown for control participants and participants with schizophrenia. Error bars denote the standard error of the mean. Deg. = degrees. ** *p* = 0.01.

To determine whether coherence thresholds differed between SZ and CON groups (Fig. 2A), data were analysed using a one-way analysis of covariance (ANCOVA) (Table 2) with IQ scores as a covariate. Motion coherence thresholds were statistically indistinguishable between control participants and those with SZ (F_(1,35)_ = 0.25, *p* = 0.62, partial-η^2^ = 0.01). Further, this held true for unfiltered data, irrespective of whether or not IQ was included as a covariate (F_(1,37)_ = 0.48, *p* = 0.49, partial-η^2^ = 0.01; F_(1,41)_ = 0.12, *p* = 0.73, partial-η^2^ = 0.003, respectively).

**Table 2:** Comparing group performance on motion coherence and equivalent noise tasks.

		F	d.f.	*p*	Partial-η^2^
**Coherence**	Th	0.25	1,35	0.62	0.01
					
**Motion**	σ_int_	3.62	1,33	0.07	0.1
	*n*_samp_	0.22	1,31	0.64	0.01
**Orientation**	σ_int_	0.20	1,33	0.70	0.01
	*n*_samp_	7.14	1,31	**0.01	0.19
**Size**	σ_int_	2.00	1,33	0.17	0.06
	*n*_samp_	2.46	1,31	0.13	0.07

Schizophrenia and control group performances were compared using a series of one-way analyses of covariance with IQ scores included as a covariate. These analyses were undertaken on filtered data. See text however for details of analyses undertaken on non-filtered (raw) data with and without IQ included as a covariate. F = F-statistic; d.f. = degrees of freedom; *p* = significance level; Partial-η^2^ = effect size; Th = motion coherence threshold; σ_int_ = internal noise; *n*_samp_ = sampling.

### Internal noise and sampling

To determine whether there was a general trend for group differences in internal noise (Fig. 2B), a multivariate analyses of covariance (MANCOVA) was undertaken with one between-participants factor (group at 2 levels: SZ and CON) and three dependent variables (orientation, motion and size internal noise), with IQ scores as a covariate. This revealed no main effect of group for internal noise (Wilks’ λ = 0.86, F_(3,31)_ = 1.63, p = 0.2, partial-η^2^ = 0.14) and held true for unfiltered data, irrespective of whether or not IQ was included as a covariate (Wilks’ λ = 0.93, F_(3,35)_ = 0.93, p = 0.43, partial-η^2^ = 0.07;Wilks’ λ = 0.87, F_(3,39)_ = 1.97, p = 0.13, partial-η^2^ = 0.13, respectively).

Similar analysis revealed a significant main effect of group on sampling (Wilks’ λ = 0.73, F_(3,29)_ = 3.57, p = 0.03, partial-η^2^ = 0.27; Fig. 2C), which held true for non-filtered data, irrespective of whether or not IQ was included as a covariate (Wilks’ λ = 0.69, F_(3,30)_ = 4.51, p = 0.01, partial-η^2^ = 0.31; Wilks’ λ = 0.61, F_(3,34)_ = 7.38, p = 0.001, partial-η^2^ = 0.39, respectively). To determine the source of this effect three *post hoc* one-way ANCOVAs were run on orientation, motion and size sampling values with one between-participants factor (group) (Table 2). Bonferroni corrections were made for three comparisons, reflecting the three visual dimensions tested (motion, orientation and size; corrected alpha = 0.02). Levels of sampling differed between the two groups (SZ and control) for the orientation task only: thus, orientation sampling was significantly lower in the SZ group (F_(1,31)_ = 7.14, p = 0.01, partial-η^2^ = 0.19). Once again, these findings held true for unfiltered data also, irrespective of whether or not IQ was included as a covariate(F_(1,32)_ = 9.6, p<0.01 partial-η^2^ = 0.23; F_(1,36)_ = 17.24, p<0.001, partial-η^2^ = 0.32, respectively).

### Correlations between psychophysical performance and clinical measures / IQ scores

To determine whether psychophysical performance was related to symptom severity a series of partial correlations were undertaken on behavioral measures and PANSS scores. These included total PANSS scores, positive, negative and general psychopathology sub-scale scores, as well as a cognitive factor (comprised of the sum of a sub-set of questions in the PANSS test), which overlaps with the concept of disorganization syndrome [45] and has been shown to predict performance on a contour integration task in SZ [16]. Following [17], we also looked for correlations between task performance and scores on question P2 of the PANSS test (conceptual disorganization; DIS). No significant correlations were found between behavioural measures and any of the PANSS scores listed (Table 3), irrespective of whether or not IQ was added as a covariate in the analyses (*p*s>0.05). Note, however, that there was relatively low variance in participants’ PANSS scores (overall scores generally indicating low-to-moderate symptoms), potentially reducing the likelihood of detecting a correlation. In addition, there was only one participant with SZ who exhibited even moderate conceptual disorganization (i.e. scoring >3 on PANSS itemP2). Neither were there any significant correlations between behavioural measures and medication dosage (CLZ equivalents) (*p*s>0.05). Finally, IQ scores were found to correlate (negatively) with coherence thresholds (*p* = 0.02), but not with internal noise or sampling (*p*s>0.05).

**Table 3:** Partial and standard bivariate correlations between psychophysical performance and clinical measures / IQ.

			Motion		Orientation		Size	
		Th	σ_int_	*n*_samp_	σ_int_	*n*_samp_	σ_int_	*n*_samp_
**tPANSS**	**R**	0.07	-0.20	-0.26	0.06	0.08	-0.17	-0.03
	***p***	0.78	0.43	0.32	0.83	0.75	0.50	0.90
**tPSS**	**R**	-0.32	-0.28	-0.02	-0.01	0.03	0.18	0.13
	***p***	0.21	0.28	0.95	0.96	0.92	0.48	0.62
**tNSS**	**R**	0.23	-0.03	-0.39	0.15	-0.09	-0.43	-0.18
	***p***	0.37	0.90	0.13	0.56	0.73	0.09	0.50
**tGSS**	**R**	0.15	-0.22	-0.21	0.01	0.22	-0.13	-0.01
	***p***	0.57	0.40	0.42	0.98	0.41	0.62	0.97
**tDIS**	**R**	0.13	-0.05	-0.25	0.21	-0.16	-0.27	-0.26
	***p***	0.61	0.85	0.33	0.42	0.53	0.30	0.32
**DIS**	**R**	0.07	0.10	-0.13	0.05	-0.08	-0.10	-0.07
	***p***	0.79	0.71	0.63	0.84	0.76	0.70	0.79
**CLZ**	**R**	-0.32	-0.27	0.04	0.04	0.40	-0.04	0.26
	***p***	0.21	0.30	0.89	0.89	0.12	0.88	0.32
**IQ**	**R**	-0.48	-0.25	0.19	-0.07	-0.18	0.18	0.36
	***p***	0.02	0.27	0.41	0.77	0.42	0.40	0.09

Partial correlations are shown for psychophysical and clinical variables for participants with schizophrenia whilst controlling for the effects of IQ scores (all rows except bottom two). Standard bivariate correlations are also shown for psychophysical measures and IQ scores (bottom two rows). R = Pearson’s correlation coefficient; *p* = significance level; Th = motion coherence threshold; σ_int_ = internal noise; *n*_samp_ = sampling. See legend to Table 1 for further details of abbreviations used for clinical measures.

## Discussion

This study was designed to test the hypothesis that impoverished integration represents a characteristic (and generalised) feature of visual processing in SZ. To this end, we tested participants (with and without SZ) on a motion coherence task and a series of discrimination tasks that probed local and global processing limits for judgements of average motion, orientation and size. We report that coherence thresholds and levels of internal noise were indistinguishable between the two groups, as were levels of sampling (a measure of global integration) for motion and size judgements. In fact, the only statistical difference in psychophysical performance between the two groups was for orientation sampling: participants with SZ typically pooled fewer samples when estimating average orientation. Consequently, we conclude that a generalised impairment in visual integration is *not* a characteristic feature of SZ, but is instead, restricted to judgements of visual orientation.

Previous reports of an orientation integration deficit in SZ have been made on the basis of impaired performance on contour detection tasks, in which the participant must detect the presence of an elongated contour -composed of discrete oriented elements (Gabors)- embedded in a field of randomly oriented distracters. In these tasks, performance at least in part, reflects the limits imposed by orientation integration, since the global contour is only revealed if orientation information is integrated across multiple individual elements. Relative to control participants, individuals with SZ are less accurate at identifying the shape or location of a contour [14–18] and are more susceptible to the disrupting effects of adding orientation jitter to its individual component elements [34,35].

However, contour detection studies do not provide an *independent* estimate of global integration since performance may be limited by additional processes. For example, participants with SZ *may* also be less precise at reporting the orientation of an isolated Gabor element [34] and exhibit abnormal patterns of contextual modulation from *nearby* flanking distracters [34,35], both of which likely contribute to impaired detection thresholds in the patient group. (See [3,46,47] however for contrary evidence of normal orientation discrimination and intact contextual processing of orientation information in SZ). Further, Schallmo et al.[35] have shown that lower IQ levels in the participants with SZ may contribute to inter-group differences in baseline detection performance: IQ scores correlated negatively with detection thresholds and differed significantly between groups. This is potentially problematic since many studies do not measure, report and/or control for intelligence.

Thus, previous studies of orientation integration in SZ have tended to confound a number of composite processes, e.g. local processing, global processing, and potentially, general cognitive factors such as IQ and attention. In contrast, we have used the EN paradigm to derive *independent* estimates of local and global processing in SZ, and have demonstrated a selective impairment in the participants’ ability to integrate orientation information. Further, we analysed the data both with and without IQ included as a covariate. Note however, that whilst ANCOVA removes the variance associated with a covariate (i.e. IQ in this case), this is not equivalent to ‘controlling for its effects [48]. Indeed, in some instances, removing variance associated with a covariate can actually create spurious effects, or else, reduce the likelihood of detecting an effect (for example, when the covariate and independent variable are closely related and represent overlapping constructs). Nonetheless, including IQ in the analyses had no effect on the results, suggesting that the findings reported are robust, and are unlikely to be driven by inter-group differences in IQ.

We think that it is similarly unlikely that the results can be explained by differences in attention between SZ and control groups. Though we have previously reported that diverted attention leads to poorer sampling for orientation averaging [49], the effects we report here were specific to the orientation judgements, and there is no reason to assume that orientation tasks make greater demands on attentional resources than judgements involving other spatial dimensions. Thus, levels of *motion* and *size* sampling did not differ between participants with and without SZ.

We included a measure of visual size averaging in our battery of tasks because, increasingly, this is the visual attribute most commonly considered in studies of voluntary averaging [50–52]. Further, in our experience, size-averaging recruits relatively higher levels of sampling than motion -or orientation- averaging (see Fig. 2), so that we can be confident that our failure to find differences between observers with and without SZ on this task cannot be attributed to a floor effect arising from generally poor/absent averaging. We are similarly confident that the absence of inter-group differences on the motion task are not due to floor effects, since reliable differences in motion internal noise and sampling have previously been reported using the same technique in a study of normal development, despite comparable sampling levels [53].

The finding that all motion perception measures were normal in our participants with SZ was somewhat unexpected. A relatively common finding in studies of vision in SZ is that motion perception is impaired (see [12] for a review), with a growing body of evidence describing elevated motion coherence thresholds [13,19–21] (see [54] however), impaired speed discrimination [24–29] and abnormal levels of motion surround suppression [55,56]. In contrast, performance is seemingly unaffected when only a single (local) direction of motion must be reported, e.g. for a drifting grating [13]. This has led some to suggest that motion processing deficits in SZ are restricted to higher-level motion-sensitive areas where local motion signals are integrated and contextual effects mediated [21], e.g. the medial temporal (MT) and medial superior temporal (MST) areas [57]. From these findings, one might expect normal levels of internal noise in SZ, coupled with reduced levels of motion sampling and elevated coherence thresholds. However, this was not the case: all psychophysical measures of motion processing were normal in the participants with SZ.

There are a number of potential explanations for this discrepancy between our own findings and previous reports in the literature. First, it is possible that our motion coherence and EN stimuli were not optimal for uncovering group differences in performance. However, this is unlikely: we used standard stimuli comprised of 100 dots, drifting at a velocity of 3 deg/sec. In the only study to parametrically manipulate element number and motion speed in parallel [54] the authors showed that the *difference* between SZ and control group coherence thresholds was maximal for a 100-dot stimulus that drifted at slow speeds. Although the authors did not test performance at speeds less than 6 deg/sec, a separate study has shown that the effect persists at 3 deg/sec [58]. Further, inter-group differences in coherence thresholds do not rely on the use of limited or infinite life-time dots in SZ [33,59]. Consequently, it is unlikely that our choice of stimulus parameters underlies the absence of an effect. It is also unlikely that we lacked statistical power, since a highly significant group difference *was* reported for orientation sampling using the same cohort of participants and experimental design; further, a number of studies have reported a group difference in coherence thresholds with a considerably smaller population sample, e.g. only 13 participants with SZ and 14 controls [20]. In addition, we have previously shown elevated motion coherence thresholds in a clinical group (migraine) using identical methods and a comparable sample size [40].

Finally, it is possible that the participants with SZ recruited in this study were unrepresentative of the population as a whole, or else, differed in some critical way to those tested in previous studies. For example, all of our participants with SZ were outpatients, whilst previous studies suggest that visual abnormalities may be more pronounced in acute / forensic inpatients [2,3,60]. Further, all of our participants were relatively high functioning, with a group average IQ that exceeded 100 and a relatively low average symptoms score (tPANSS = 58 out of a maximum of 210). Since IQ scores are known to correlate with performance on a number of visual tasks in SZ, e.g. velocity discrimination [25,28] and contour detection [35], it is possible that our chances of finding an effect would have been greater if more symptomatic patients had been included in the study. Indeed, in our own data, although coherence thresholds did not differ between groups (SZ and CON), they did correlate (negatively) with IQ scores. Given that many studies of vision in SZ do not report (or control for) differences in IQ (e.g. [20,21]), it is possible that some findings previously reported in the literature may be partially confounded by this factor. We note however that a number of studies *have* reported systematic visual abnormalities in SZ whilst controlling for IQ, e.g. [14,61].

On the topic of potential confounds, it is important to note that although all participants included in this study had a minimum visual acuity of 20/20, we did not check for systematic inter-group differences in acuity within the ‘normal’ range. Thus, acuities greater than 20/20 were not recorded: participants were simply included or excluded on the basis of whether or not they reached criterion. This is potentially relevant since a recent study (published subsequent to our data collection phase) has suggested that individuals with acuities greater than 20/20 exhibit lower contrast detection thresholds and superior contour integration performance than participants with 20/20 vision [62]. Further, it has been suggested that individuals experiencing psychosis may exhibit poorer visual acuity [63,64], putatively because, relative to non-affected controls, they are less likely to monitor and attend to their physical health needs [64]. Nonetheless, we believe it unlikely that the two groups studied here (SZ and control) differed significantly with respect to visual acuity, since they had indistinguishable levels of internal noise and basic spatial offset thresholds (*ps*<0.05, data not shown). These two measures essentially measure the participant’s ability to undertake fine spatial discriminations, and as such, would be expected to differ if there were systematic differences in acuity between the groups. Further, it is not clear why orientation (rather than size) would be systematically affected. Nonetheless, we believe that in light of these findings [62], future studies of visual perception in SZ should measure and compare visual acuity as standard practice alongside other relevant measures (e.g. IQ and symptoms scores).

Another potential difference between our own patients and those used in previous experiments is with respect to their level of exposure to psychophysical testing. All of our participants with SZ were recruited from an outpatient clinic at the Institute of IoP, many of whom have been tested previously on visual tasks and, as a consequence, were practiced psychophysical observers at the time of testing. Although we are not aware of a previous motion perception study undertaken with this particular patient cohort, there is some evidence that motion processing thresholds may benefit from general (procedural) learning. In one study of motion coherence, thresholds fell with practice, an effect that was more pronounced for a group of participants with SZ than for a matched control group [59]. By the end of five training sessions, patients had undergone a 47% improvement in performance, such that their coherence thresholds did not differ from the control participants’. Further, there was a trend (p = 0.05) for this learning effect to transfer to a different (untrained) task, which involved judgements of relative velocity. Consequently, our use of experienced psychophysical observers may also have reduced the likelihood of finding a group difference in performance. However, it is worth noting that evidence for the efficacy of cognitive training in SZ is mixed (see [65] for a review), particularly with respect to issues of transfer and generalisation of learning (e.g. [66]). Further, our control group was made up of a similar mix of psychophysically experienced and psychophysically naïve observers.

Finally, there is a possibility that the discrepancy between our findings and previous reports reflects, in part, a reporting bias in the literature. Thus, according to the ‘file-drawer’ problem [67], there is a tendency for negative results to go unpublished. Consistent with this possibility, whilst a number of studies have reported elevated coherence thresholds in SZ [13,19–21], we are not alone in obtaining a null result. In a recent study of 29 participants with SZ and 23 without, motion coherence thresholds were found to be indistinguishable between the two groups [54]. Further, in the same study, the participants with SZ showed elevated contour integration thresholds (relative to a control group), arguing against this particular population sample being unrepresentative. (Indeed, one wonders if these negative findings would have been published at all in the absence of significant inter-group differences in contour integration performance). It is therefore possible that elevated motion coherence thresholds in SZ are contingent on a moderating variable that is at yet unidentified.

A critical question that arises from our findings is why the visual averaging deficit that we report is not generalised, but is instead restricted to a *specific* visual dimension. Thus, a number of models of anomalous perception in SZ posit abnormalities in fundamental cortical processes that are replicated across multiple brain regions [9,68], such that one might expect *all* visual dimensions to be affected. Indeed, studies of SZ have reported perceptual abnormalities across a wide range of visual dimensions and task types (see [1] for a review). However, these findings reflect data that have been gathered across multiple research groups, using distinct diagnostic criteria, patient cohorts and experimental designs, such that they cannot be compared directly. Further, many of these studies cannot distinguish between localised abnormalities in defined neural networks (e.g. integration of motion signals in area MT) and more general effects driven by inter-group differences in, for example, attention, motivation or task comprehension. For this reason, more informative studies are those in which a single cohort of participants is tested across a range of visual dimensions.

Where studies of visual processing in SZ *have* tested a single group of participants across multiple visual dimensions (of which there are few to date), the findings typically implicate selective abnormalities on a subset of tasks, rather than a generalised, i.e. widespread, impairment. For example, when Tibber and colleagues [3] had individuals with and without SZ judge the brightness, contrast, orientation and size of targets embedded in high-contrast contextual surrounds, they found that participants with SZ exhibited abnormal responses (reduced biases) for judgements of contrast and size only, i.e. orientation and brightness judgements were unaffected. Similarly, in a study involving judgements of visual brightness, contrast, orientation, size *and* motion (in the context of high-contrast surrounds), Yang and colleagues [46] reported a reduced bias in SZ for judgements of contrast only, i.e. all other judgements were normal. Although there is some inconsistency in the findings of these two studies (i.e. with respect to size judgements, see discussion in [3] however), they nonetheless reinforce the findings reported here, and implicate a selective impairment in a *subset* of cortical networks.

With respect to why *orientation* averaging is ‘special’, i.e. why it is selectively impaired in SZ whilst motion and size averaging are not, we can only speculate. Although this pattern of findings cannot be captured by a simple model of, for example, selective impairment in cortical versus pre-cortical [3] loci, or magno-cellular versus parvo-cellular [69] pathways, we cannot rule out the possibility that it reflects more localised differences in the underlying cyto-architecture or receptor / neurotransmitter distribution of implicated cortices [70]. Thus, it is likely that perceptual processes underlying responses to different versions of the equivalent noise task are subserved by largely distinct (potentially dimension-specific) neural networks [71], and that these may be differentially susceptible in SZ. In support of this, there is robust evidence that whilst pooling of motion signals takes place in higher motion processing areas such as area MT and MST [57], the integration of orientation signals is mediated (in part) by lateral connections *within* relatively early orientation-selective visual areas, e.g. area V1 [72]. Consequently, one hypothesis that has the potential to explain a *selective* deficit in orientation pooling in SZ is reduced intra-cortical connectivity in these early visual cortices. Thus, if the spatial extent of visual pooling were reduced in SZ, orientation averaging judgements would be affected, whilst motion averaging judgements might be spared, since the latter rely on distinct (and dedicated) higher-level areas.

In support of reduced patterns of connectivity in early visual areas, Anderson and colleagues [73] have shown that, relative to controls, population receptive field sizes in SZ (a measure of local intra-cortical connectivity) are reduced in early visuo-cortical areas (V1-V3). Further, this difference is only significant in area V1, the earliest stage in the visual processing hierarchy where orientation information is extracted. Impaired patterns of connectivity in early visual areas within SZ is also consistent with a number of other psychophysical observations in the literature, e.g. reduced suppression [2], facilitation [74] and crowding [34] effects from contextual cues / distracters in a stimulus. In addition, levels of GABA (the brain’s primary inhibitory neurotransmitter) are reduced in area V1 in SZ (relative to controls) and correlate positively with a measure of orientation-tuned surround suppression [65]. Taken together, these studies suggest that patterns of intra-cortical connectivity are severely disrupted in early visual areas in SZ, a finding that might explain the selective deficit in orientation averaging reported here.

In conclusion, the findings reported suggest that impaired visual integration does *not* represent a generalised feature of SZ. Instead, they support previous studies, which indicate that distinct global integration and averaging tasks are sub-served by largely independent mechanisms and cortical loci [47,71,75,76], which may be differentially susceptible to acquired damage / developmental abnormality. Consequently, to speak of a general deficit in global processing may represent too coarse a generalisation. In the data we report, relative to matched control participants, all psychophysical measures recorded were normal in the participants with SZ, with the single exception of orientation sampling: the participants with SZ typically pooled fewer local orientation estimates when reporting average orientation (EN analysis). The fact that this effect could be detected in a relatively high functioning outpatient group that included experienced psychophysical observers, and in the context of normal performance on closely-matched motion and size tasks, suggests that orientation processing may be particularly susceptible to impairment in SZ. One possible explanation for these findings is that patterns of intra-cortical connectivity are disrupted in early visual areas in SZ, a notion that is supported by a recent study involving population receptive field mapping. Nonetheless, future studies should be undertaken to test this hypothesis explicitly, and further, to determine to what extent (if any) this relates to other visuo-perceptual abnormalities in SZ such as impaired contour integration.

## Supporting Information

S1 Dataset Supporting information. (XLSX)
