# Correction: Local and global limits on visual processing in schizophrenia

**DOI:** 10.1371/journal.pone.0123801

**Published:** 2015-03-30

**Authors:** The PLOS ONE Staff

An affiliation for the sixth author is missing. Steven Dakin is affiliated with: Institute of Ophthalmology, University College London and the Department of Optometry & Vision Science, University of Auckland, Private Bag 92019, Auckland 1142, New Zealand.
